# Perception and Impact of the Family Adoption Program (FAP) Among Indian Medical Students: Benefits and Challenges

**DOI:** 10.7759/cureus.73893

**Published:** 2024-11-18

**Authors:** Praveen Ganganahalli, Sandeep G Yankanchi, Mallikarjun Yadavannavar, Rekha Udgiri

**Affiliations:** 1 Community Medicine, Shri. B. M. Patil Medical College, Hospital and Research Centre, BLDE (Deemed to be University), Vijayapura, IND; 2 Preventive Medicine, Shri. B. M. Patil Medical College, Hospital and Research Centre, BLDE (Deemed to be University), Vijayapura, IND

**Keywords:** benefits, challenges, communication, fap, undergraduate students

## Abstract

Background

The National Medical Commission of India recommends the Family Adoption Program (FAP) under Competency-Based Medical Education (CBME) in the MBBS curriculum. Medical students can witness firsthand the living situations of the people they treat as patients in the hospital through community participation. Students also learn how various health factors impact patients in real-world settings. This study aimed to assess the perceived benefits and impact of FAP in the educational and professional development of medical students and explore the challenges and critical facilitators for medical students participating in FAP.

Methodology

First, second, and third-year medical undergraduate students who were enrolled in FAP by the Department of Community Medicine participated in an observational study during the last visit of FAP by using a structured questionnaire on the perception of the benefits of FAP and its possible future impact, as well as an investigation of the obstacles and enablers encountered when conducting FAP.

Results

Approximately 90% of the 305 students who participated believed that FAP is beneficial for their own personal and professional growth. Additionally, they mentioned that faculty engagement was the main factor that encouraged them to participate in FAP, with the benefits outweighing the challenges.

Conclusions

Medical students often find it difficult to get involved in family adoption programs, but they can be helped by a friendly environment, the correct tools, and mentorship. Medical schools can encourage a future generation of healthcare providers who are more involved and empathetic by addressing these issues and strengthening essential facilitators.

## Introduction

Medical students can get their firsthand experience of the living situations of the people they treat as hospital patients through community participation. Students can also understand how different health variables affect patients in the real world. The community that the institute has adopted through medical students gain experience from the specialization of Community Medicine [1].

According to the National Medical Commission (NMC) 2022 guidelines [2], MBBS students are required to participate in the Family Adoption Program (FAP). At least three to five families should be assigned to each student, and they must build rapport, understand health-related factors, and help improve the healthcare of both families and the community. It is, therefore, anticipated to aid in achieving universal health coverage [2,3].

In a community-oriented medical education model, the community serves as the learning environment within a particular social context. Engaging in educational experiences pertinent to community needs is a proactive endeavor for educators, students, and community members. Medical education centered on community health needs is successful in helping students comprehend the medico-social determinants of health, according to the World Health Organization and the World Federation for Medical Education. Community settings offer comprehensive learning, unlike hospital-based teaching, which is the norm [4].

The FAP seeks to promote health equity by providing Indian medical graduates with practical experience in community-based healthcare. The NMC’s strategy for FAP seeks to advance equitable and universal healthcare, guarantee the availability of qualified professionals across the country, improve access to high-quality medical education, and foster a community health perspective to support national health goals. The FAP also seeks to enhance students’ communication abilities, foster empathy for rural and adoptive families, foster leadership in the delivery of healthcare, establish accountability as primary consultants, and impart fundamental clinical skills [5,6].

This study would contribute to understanding how FAP can be integrated into medical education in India, potentially fostering a culture of community engagement and social responsibility among future healthcare professionals.

## Materials and methods

Phase 1, Phase 2, and Phase 3 Part I medical undergraduate students enrolled in the FAP by the Department of Community Medicine participated in this observational study. Informed consent and Institutional Ethics clearance were obtained before data collection. A convenience sampling technique was used to enroll the students in the study using the pre-defined inclusion criteria. The study involved students from all three years of the medical course who were present during the visit. Students who were absent during the last visit were excluded from the study.

Data were collected using a structured questionnaire (mentioned in Appendices) via a Google Forms link. The data were collected from January to August 2024 during the last visit to the FAP by the students in their respective years. The questionnaire covered sociodemographic data, perception of the benefits of FAP and its possible future impact, as well as an investigation of the challenges faced and enablers encountered while conducting FAP.

The students were asked to mark the appropriate scale for perception toward FAP using a three-point Likert scale (Agree, Neutral, Disagree). In contrast, the perception of the students of the possible impact of FAP was measured using scales of positiveness and satisfaction and by scoring from 1 (challenges outweigh benefits) to 5 (benefits outweigh challenges). The students were asked to mention the challenges and key facilitators for medical students participating in FAP in multiple answers by using open-ended questions. Data were analyzed using SPSS version 27 (IBM Corp., Armonk, NY, USA) to assess frequency distributions and chi-square tests to determine the significance of relationships between variables.

## Results

**Figure 1:**
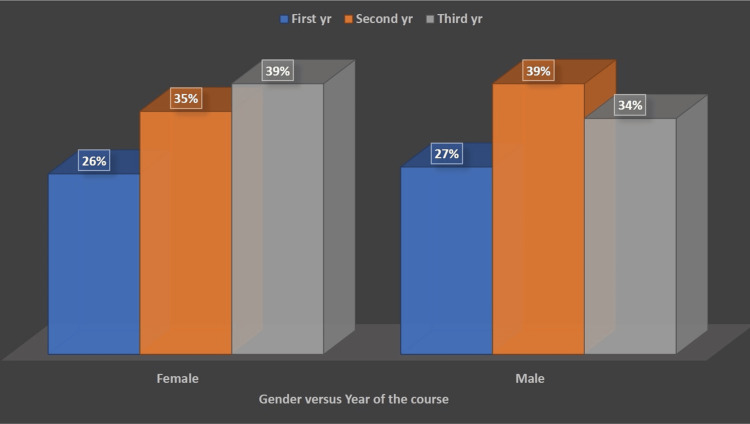
Distribution of students according to the gender and year of the course (n = 305).

A total of 305 students from all three years responded to the questionnaires sent to them through the WhatsApp group. Among them, 29% (89) were from the first professional year, 30% (93) from the second professional year, and 40% (123) from the third professional year. Overall, 59% (181) were female students, whereas 41% (124) were male students (Figure 1).

Students reported a favorable impression of the FAP following their yearly visit. Table 1 demonstrates that the majority of students strongly agreed or agreed with the positive components of the FAP, while very few (none) expressed disagreement or neutrality. Students believed that FAP improved their understanding of the role that families play in health and disease, improved their communication skills, helped them create empathy for patients by assisting them to comprehend the impact, and explained how working collaboratively with other organizations can enhance the health of families and the community. Approximately 80.9% (247) of students said that FAP improved their level of satisfaction with medical school in general.

**Table 1:** Students’ perception of the benefits of FAP (n = 305). FAP: Family Adoption Program

Perception	Variable	First year (n = 89)	Second year (n = 93)	Third year (n = 123)	Total (n = 305)	Chi-square (p-value)
FAP has improved my understanding of patient-family dynamics	Agree	83 (93%)	82 (88%)	112 (91%)	277 (90.8%)	9.904 (0.27)
	Neutral	06 (6.7%)	09 (9.6%)	09 (7.3%)	24 (7.8%)	
	Disagree	00 (0%)	02 (2.1%)	02 (1.6%)	04 (104%)	
FAP has enhanced my communication skills with patients and their families	Agree	82 (92%)	81 (87%)	108 (87.8%)	271 (88.8%)	6.318 (0.60)
	Neutral	07 (7.8%)	10 (9.3%)	13 (10.5%)	30 (9.8%)	
	Disagree	00 (0%)	02 (2.1%)	02 (1.6%)	04 (1.4%)	
FAP has helped me manage stress related to patient care and medical training	Agree	76 (85%)	63 (67.7%)	92 (74.7%)	231 (75.7%)	15.73 (0.04)
	Neutral	12 (13.4%)	24 (25.8%)	28 (22.7%)	64 (20.9%)	
	Disagree	01 (1.1%)	06 (6.4%)	03 (2.4%)	10 (3.2%)	
FAP has increased my empathy toward patients and their families	Agree	81 (91%)	79 (85%)	115 (93%)	275 (90.1%)	17.67 (0.02)
	Neutral	07 (7.8%)	13 (14%)	07 (5.6%)	27 (8.8%)	
	Disagree	01 (1.1%)	01 (1%)	01 (0.8%)	03 (0.9%)	
FAP has provided me with practical skills applicable to my medical practice	Agree	81 (91%)	74 (80%)	109 (88.6%)	264 (86.5%)	13.81 (0.08)
	Neutral	08 (9%)	18 (19%)	12 (9.7%)	38 (12.4%)	
	Disagree	00 (0%)	01 (1%)	02 (1.6%)	03 (0.9%)	
FAP has improved my ability to collaborate with other healthcare professionals	Agree	79 (88.7%)	72 (77%)	98 (79.6%)	249 (81.6%)	8.574 (0.38)
	Neutral	10 (8.3%)	18 (19%)	21 (17%)	49 (16%)	
	Disagree	02 (2.2%)	03 (3.2%)	04 (3.2%)	09 (2.9%)	
FAP has facilitated my personal growth and self-awareness	Agree	81 (91%)	78 (83%)	109 (88.6%)	268 87.8(%)	5.827 (0.66)
	Neutral	07 (7.8%)	13 (14%)	13 (10.5%)	33 (10.8%)	
	Disagree	01 (1.1%)	02 (2.1%)	01 (0.8%)	04 (1.4%)	
FAP has positively influenced my academic performance	Agree	77 (86.5%)	67 (72%)	83 (67%)	227 (74.4%)	10.454 (0.23)
	Neutral	17 (19%)	20 (21.5%)	31 (25%)	68 (22.2%)	
	Disagree	01 (1.1%)	06 (6.4%)	09 (7.3%)	16 (5.2%)	
FAP has improved my overall satisfaction with medical education	Agree	80 (89.8%)	70 (75%)	97 (78.8%)	247 (80.9%)	9.699 (0.28)
	Neutral	08 (8.9%)	21 (22.5%)	24 (19.5%)	53 (17.3%)	
	Disagree	01 (1.1%)	02 (2.1%)	02 (1.6%)	05 (1.6%)	
I would recommend FAP to other years of medical students	Agree	80 (89.8%)	69 (74%)	105 (85%)	254 (83.2%)	14.814 (0.06)
	Neutral	09 (10%)	20 (21.5%)	14 (11%)	43 (14%)	
	Disagree	00 (0%)	04 (4.3%)	04 (3.2%)	08 (2.6%)	

Table 2 illustrates how positively 197 (64.5%) students felt the FAP would affect their future professional and educational development. Approximately 55% (168) of the students rated the benefits as outweighing the problems with a score of 4 or above, whereas 24% (73) of the students felt that the challenges were more significant in FAP than the benefits. In general, 69.8% (213) of the students expressed satisfaction with their FAP experience.

**Table 2:** Perception of the students about the impact of FAP (n = 305). FAP: Family Adoption Program

Perception	Variables	First year (n = 89)	Second year (n = 93)	Third year (n = 123)	Total (n = 305)	Chi-square
How do you perceive the impact of FAP on your ability to provide patient-centered care?	Positive	63 (70.7%)	59 (63%)	75 (61%)	197 (64.5%)	4.295 (0.83)
	Neutral	22 (24.7%)	30 (32%)	40 (32.5%)	92 (30.1%)	
	Negative	04 (4.4)	04 (4.3%)	08 (6.5%)	16 (5.2%)	
How do you perceive the balance between the benefits of participating in FAP and the challenges you face?	Scale - 1 = Challenges outweigh benefits	06 (6.7%)	13 (13.9%)	06 (4.8%)	25 (8.1%)	9.104 (0.33)
	Scale - 2 = Challenges little outweigh benefits	12 (13.5%)	12 (12.9%)	26 (21%)	50 (16.3%)	
	Scale - 3 = Challenges equals benefits	19 (31%)	17 (18.2%)	24 (19.5%)	60 (19.6%)	
	Scale - 4 = Benefits little outweigh challenges	33 (37%)	34 (36.5%)	45 (36.5%)	112 (36.7%)	
	Scale - 5 = Benefits outweigh challenges	19 (31%)	17 (18.2%)	22 (17.8%)	58 (19%)	
Overall, how satisfied are you with your experience in the FAP?	Satisfied	74 (83%)	57 (61.2%)	82 (66.6%)	213 (69.8%)	14.104 (0.07)
	Neutral	14 (15.7%)	30 (32.2%)	34 (27.6%)	78 (25.5%)	
	Not satisfied	01 (1.1%)	06 (6.4%)	07 (5.6%)	14 (4.5%)	

Table 3 illustrates the difficulties that the students encountered while participating in the FAP. These difficulties included time limits, language problems, communication breakdowns, persuading the families of the visit’s goal, and sociocultural barriers to information dissemination. On the other hand, the students identified the following as essential facilitators in overcoming the obstacles and igniting their interest in the FAP: flexible participation, departmental involvement, helpful mentors, and planned schedules to lessen the workload.

**Table 3:** Challenges and key facilitators for medical students in participating in FAP (n = 305) (multiple answers). FAP: Family Adoption Program

Challenges	Frequency, n = 305 (%)	Key facilitators	Frequency, n = 305 (%)
Time constraints	170 (56%)	Supportive faculty/mentors	265 (87%)
Emotional burden	64 (21%)	Interdisciplinary collaboration	168 (55%)
Lack of experience	70 (23%)	Structured or planned schedule	232 (76%)
Institutional barriers	30 (10%)	Orientation session	244 (80%)
Language barriers	183 (60%)	Flexible participation	207 (68%)
Resistance from family members	103 (34%)	Friendly behavior of the family	174 (57%)
Convincing the family reg the purpose of the visit	100 (33%)	Field experience	137 (45%)
Extreme heat	46 (15%)	Communication with family	131 (43%)
Availability of family members during the visit	33 (11%)	Activities like environment protection, roleplay, and health education	235 (77%)

## Discussion

Approximately 90% of the 305 students surveyed felt that FAP would help them advance both personally and professionally. Furthermore, they stated that the primary motivator for their participation in FAP was teacher engagement and that the benefits outweighed the challenges.

According to Landge et al. [6], 92 of the 100 students who were enrolled in FAP completed the feedback form via a Google link with a 1:1 male-to-female ratio. Overall, 80% of the 92 students who responded indicated they would like to continue participating in the FAP during their professional careers and that they believed it was a terrific experience. The practical implementation of FAP requires thorough planning, robust sensitization, intersectoral collaboration, and prior training.

Padival [7] analyzed students’ reflections on the FAP experience and came to the following conclusion: “This first part of my family adoption experience will be among the most memorable of my time as a medical student. It provided me with background information about the patients I would subsequently visit in my clinical postings throughout the ensuing years. Although unsure how the visits would benefit families and society, they undoubtedly made me a more conscious medical graduate.”

Patra et al. [8] studied how students perceived the FAP and discovered that while most students (96.3%) were able to explain to the family why they were there, very few were able to get all the information, even during follow-up visits. Obstacles to communication and language, trust issues with the students, family disinterest, and insufficient student understanding were the key challenges that needed to be addressed for FAP to be successful.

According to Basagoudar et al. [9], of the 172 first-year students, 33.1% were female, and 66.9% were male. Overall, 66.9% of the students said that FAP was a very positive experience. Of the research participants, 55.2% expressed satisfaction with their new family. Over their professional years, most students expressed interest in participating in such activities. Careful planning, intersectoral coordination, robust sensitization, and training initiatives are necessary for the successful implementation of FAP. In a study, Arora et al. [10] concluded that family adoption is instrumental in improving self-perceived communication skills and the ability to help the community with their health problems.

According to Arumugam et al. [11], there should be a strategy to make students aware of community health care from the moment they begin working as professionals. Vaniker et al. [12] stated in their editorial that the FAP has the potential to raise undergraduate medical education to new heights and pave the way for a healthier and more empowered nation. It also gave the students a chance to interact and hone their communication skills with the general public, with the potential to aid them in their future practice.

In their study on environmental wellness in FAP, Sahoo et al. [13] reported that while all students were aware of environmental health, female students had better behaviors. By implementing these effective practices for environmental wellness, they can raise awareness of the issue in the community and eventually gain the competency needed for field visits. This will assist them in influencing the community’s perception of the environment’s significance for health and wellness.

After performing a SWOC (Strengths, Weaknesses, Opportunities, and Challenges) analysis of the FAP experiences, Shikha et al. [14] concluded that to keep the family from becoming orphaned, another student should continue the adoption cycle after the departing student has completed their studies and offer continuum care services.

According to Raja et al. [15], the biggest obstacle to the implementation of FAP is that some pupils are uninterested. Few students participate entirely. During their first year of medical college in India, students from different states find it difficult to interact in other languages with rural populations. In addition to finding it difficult to adjust, students from other cultures and customs are often unaware of the local way of life.

There is a shortage of literature in this field of study, and this study is need of the hour and necessary to execute the new curriculum. However, student perceptions following their exposure to FAP can change and are subject to alteration based on a variety of factors, which is a limitation of this study.

## Conclusions

The majority of students had positive thoughts about the FAP, which significantly impacted their academic and professional growth and their capacity to understand patients’ viewpoints regarding health and illness. Although participating in FAP can be challenging, proper support, resources, and guidance can help students succeed. By addressing these problems and bolstering key facilitators, medical schools can promote a more engaged and compassionate generation of healthcare professionals in the future.

## Appendices

**Table 4:** Questionnaire used for data collection (Proforma).

Age (in years)			
Gender	Male	Female	
Area of residence	Rural	Urban	
Please rate the following statements based on your experience with the Family Adoption Program (FAP): tick in (√) appropriate boxes			
Perception	Agree	Neutral	Disagree
FAP has improved my understanding of patient-family dynamics			
FAP has enhanced my communication skills with patients and their families			
FAP has helped me manage stress related to patient care and medical training			
FAP has increased my empathy toward patients and their families			
FAP has provided me with practical skills applicable to my medical practice			
FAP has improved my ability to collaborate with other healthcare professionals			
FAP has facilitated my personal growth and self-awareness			
FAP has positively influenced my academic performance			
FAP has improved my overall satisfaction with medical education			
I would recommend FAP to other medical students.			
Impact	Tick appropriately		
How do you perceive the impact of FAP on your ability to provide patient-centered care?	Positive	Neutral	Negative
Overall, how satisfied are you with your experience in the Family Adoption Program (FAP)?	Satisfied	Neutral	Dissatisfied
Whether the following objectives of the FAP fulfilled?	Agree	Neutral	Disagree
How do you perceive the balance between the benefits of participating in FAP and the challenges you face? (select appropriate scale)	Scale - 1 = Challenges outweigh benefits		
	Scale - 2 = Challenges little outweigh benefits		
	Scale - 3 = Challenges equals benefits		
	Scale - 4 = Benefits little outweigh challenges		
	Scale - 5 = Benefits outweigh challenges		
What are the main challenges you have faced in participating in FAP?			
What key factors have facilitated your participation in FAP?			
